# Supplementary material for: Chemical and Mechanical Properties of Metakaolin-Based Geopolymers with Waste Corundum Powder Resulting from Erosion Testing
Source: Polymers (Basel). 2022 Nov 23;14(23):5091. doi: 10.3390/polym14235091 (PMC9739097; doi:10.3390/polym14235091)
Supplement: Supplementary file 1 [file polymers-14-05091-s001.zip › polymers-2005774-supplementary.pdf]

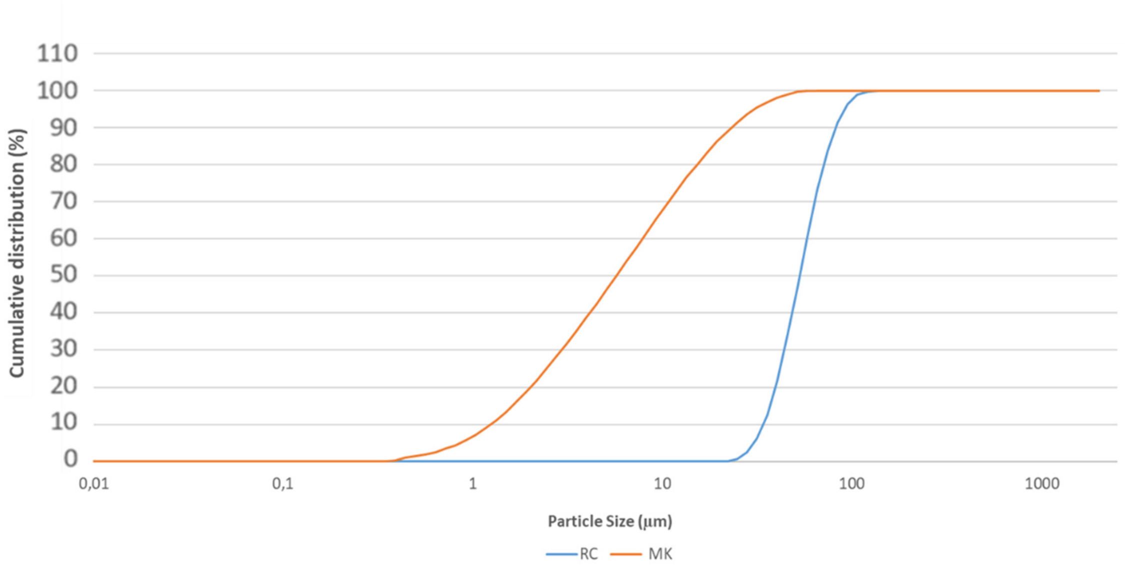

Figure S1. Comparison of particle size distribution curves of pure metakaolin (MK) and the as-received fine recycled corundum powder (RC).
